# A new taxon within *Biscutella laevigata* L. (Brassicaceae) endemic to calamine areas in southern Poland

**DOI:** 10.3897/phytokeys.160.53937

**Published:** 2020-09-08

**Authors:** Małgorzata Wierzbicka, Maria Pielichowska, Olga Bemowska-Kałabun, Adam Rostański, Paweł Wąsowicz

**Affiliations:** 1 Faculty of Biology, University of Warsaw, Miecznikowa 1, 02-096 Warszawa, Poland University of Warsaw Warsaw Poland; 2 The Maria Grzegorzewska University, Szczęśliwicka 40, 02-353 Warszawa, Poland The Maria Grzegorzewska University Warsaw Poland; 3 Faculty of Natural Sciences, Institute of Biology, Biotechnology and Environmental Protection, University of Silesia, Jagiellońska 28, 40-032 Katowice, Poland University of Silesia Katowice Poland; 4 Icelandic Institute of Natural History, Borgir vid Nordurslod, 600 Akureyri, Iceland Icelandic Institute of Natural History Akureyri Iceland

**Keywords:** Biscutella, Biscutella laevigata subsp. woycickii, Brassicaceae, new subspecies, southern Poland, taxonomy

## Abstract

A new taxon Biscutella laevigata subsp. woycickii (Brassicaceae) is described from southern Poland. The taxon is similar to B. laevigata subsp. gracilis, but differs in having thin, light-green rosette leaves very densely covered by simple non-glandular trichomes, smaller seeds and the ability to tolerate and accumulate high amounts of heavy metals. This new taxon is supported by results of cultivation experiments, as well as genetic and paleobotanical evidence.

## Introduction

Heavy-metal-rich calamine soils have been attracting human attention for several thousand years. Initially, the total area occupied by these environments was limited to small, isolated outcrops of ore-bearing rocks. Usually these areas were easy to spot due to unique vegetation covering soils that naturally developed on a metal-rich rocky substrate. Mining activities carried out in Europe since the Bronze Age (ca. 3 kyr BP) (Coulson 2012) contributed to a significant increase in the areas covered by calamine soils (nowadays represented mainly by mining waste heaps, riparian areas along rivers polluted by wastewater and sediments from ore-processing factories, mines etc.) and to the almost total destruction of the primary habitats of calamine vegetation (Baker et al. 2010). However, this huge, anthropogenic environmental change did not lead to the total extinction of all the plant taxa connected with calamine areas (Baker et al. 2010). On the contrary, while primary habitats of the calamine flora were destroyed, new and often more spacious environments have developed around the mining sites. Heaps of waste materials from early metal mines, which could not be easily colonised by other plants due to the very high content of zinc and lead, became ideal refugia for calamine-adapted plants.

Heavy-metal-polluted calamine soils and natural processes that resulted in the development of metal-tolerant vegetation covering such places have been at the focus of attention for many scholars since the beginning of the 20^th^ century (Baker et al. 2010). Soon this research demonstrated that calamine areas host unique plant taxa that are often endemic. In Europe, a number of such taxa have already been formally recognised. Examples of these taxa include: *Viola calaminaria* (DC. ex Ging.) Lej., Noccaea caerulescens (J.Presl & C.Presl) F.K.Mey. subsp. calaminaris (Lej.) Holub and Armeria alpina Willd. subsp. halleri (Wallr.) Nyman.

In the vicinity of Olkusz (southern Poland), there is an old mining area with lead and zinc mining activities dating back to the 12^th^ century (Molenda 1984). Until the end of the 20^th^ century, open, shallow pits were used to excavate Zn-Pb ores that were located close to the ground level (Szarek-Łukaszewska et al. 2015). During the 900 years of ore exploitation, natural sites of calamine vegetation have been completely erased, but the rich and diverse calamine flora can be found in secondary habitats in the vicinity of Olkusz. Calamine grasslands occurring there on old mining waste heaps have been studied by biologists since the beginning of the 20^th^ century (Wóycicki 1913).

In this paper, we argue that a new taxon endemic to calamine areas close to Olkusz in southern Poland deserves formal recognition at a subspecies level within *Biscutella laevigata* L.

## Materials and methods

The study is based on field surveys, laboratory studies including experiments under controlled growing conditions, as well as on genetic analyses. Results of these studies have already been published in several papers dealing with ecology and physiology of *B. laevigata* from calamine areas. Detailed descriptions of experiments carried out by us can be found especially in papers by Wierzbicka and Pielichowska (2004) and Wasowicz et al. (2014). We also undertook an extensive review of literature and all relevant data from already published studies are also cited in our paper.

## Taxonomy

### Biscutella laevigata L. subsp. woycickii

Taxon classificationPlantaeBrassicalesBrassicaceae

M.Wierzb, Pielich. & Wasowicz subsp. nov.

AEBBC13A-27A0-5169-B51A-37134F3D2FB9

urn:lsid:ipni.org:names:77211421-1

Figure 1

#### Type.

Poland. Olkusz, 1922, R. Kobendza, s.n. (holotype, WA0000071422 !).

#### Diagnosis.

Biscutella laevigata subsp. woycickii is similar to subsp. gracilis, but differs from the latter in having thin, light-green rosette leaves very densely covered by simple non-glandular trichomes. Plants belonging to subsp. woycickii have smaller seeds and are characterised by the ability to tolerate and accumulate high quantities of heavy metals.

**Figure 1.**
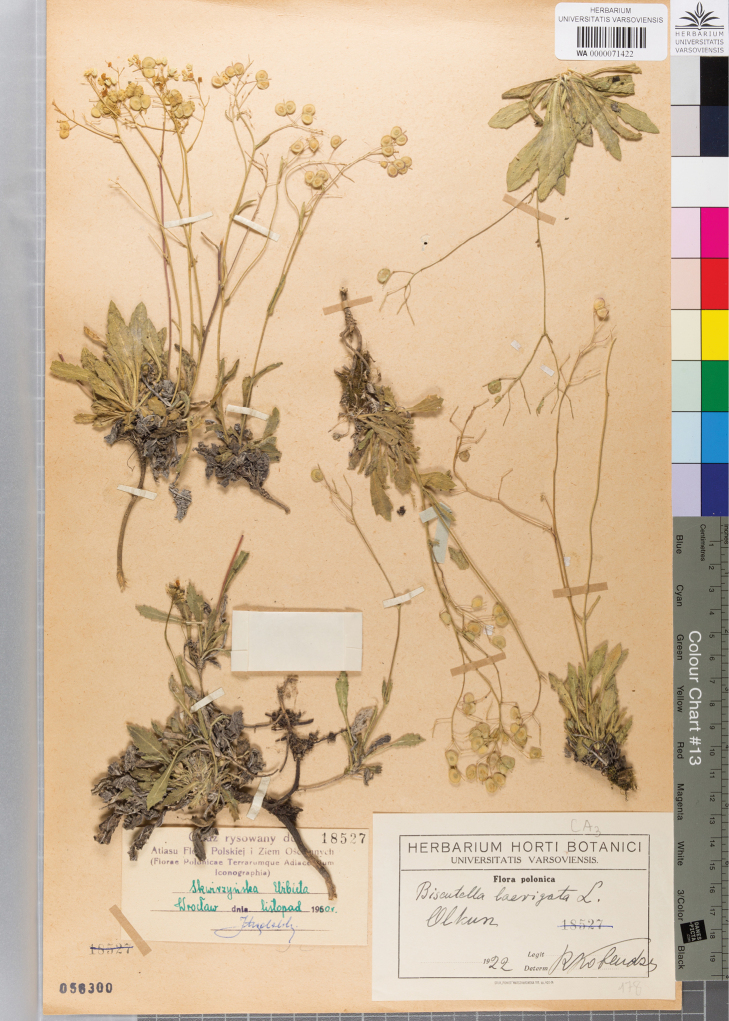
A holotype of Biscutella laevigata subsp. woycickii Wierzbicka, Pielichowska & Wasowicz (WA0000071422).

#### Etymology.

This subspecies is dedicated to a renowned Polish botanist Zygmunt Wóycicki (1871–1941), a pioneer of biological research on calamine areas.

#### Distribution and ecology.

Calamine areas in the vicinity of Olkusz, Powiat Olkuski (Olkusz County), Województwo Małopolskie (Lesser Poland Voivodeship/Province), southern Poland.

#### Phenology.

Flowering in April–May, fruiting in July–August.

#### Chromosome number.

A study carried out by Skalińska (1950) on plants from the calamine population in the vicinity of Olkusz resulted in the diploid chromosome number, 2*n* = 18.

#### Preliminary conservation status.

Currently, the taxon is known only from calamine areas in the vicinity of Olkusz, where it is quite abundant on calamine soils. The extent of occurrence (EOO) of the taxon is 7 km^2^ and the area of occupancy (AOO) is 14 km^2^. A steady decline in population size has been observed during the last 20 years. It seems that the new taxon could be classified as Vulnerable according to the IUCN criteria (Standards IUCN 2019), but more research is needed to estimate the number of mature individuals and population dynamics.

## Discussion

**Figure 2.**
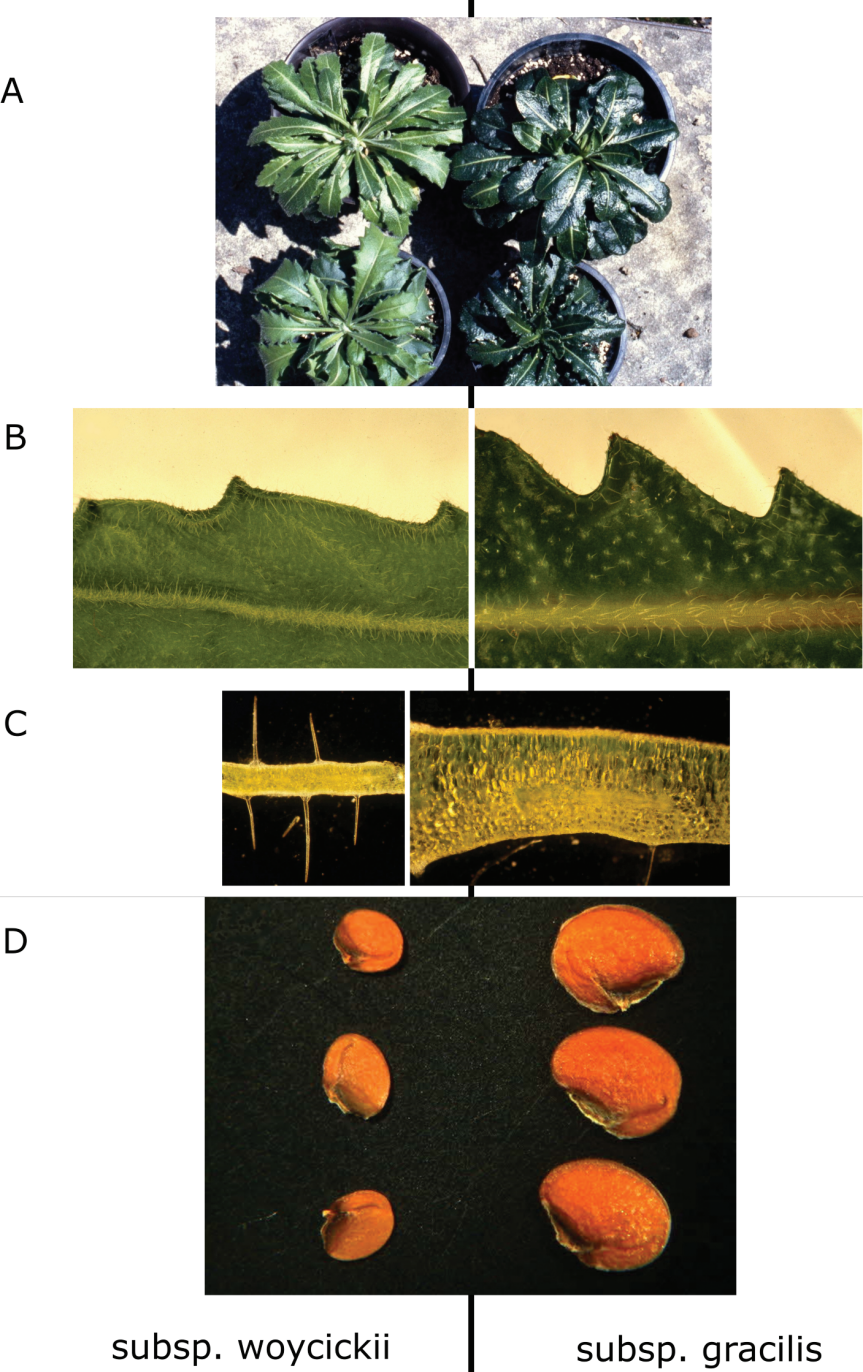
Comparison of morphological characters in Biscutella laevigata subsp. woycickii subsp. nov. and B. laevigata subsp. gracilis: **A** rosette leaves **B** leaf trichomes (both images at the same scale) **C** cross-section of the leaf blade (both images at the same scale) **D** seeds.

The morphological and geographic distinctiveness of *B. levigata* populations from the Olkusz Ore Bearing Region have been recognised by botanists already in the 19^th^ century (Zalewski 1886, Wóycicki 1913, Zając 1996), but the detailed morphological, anatomical and physiological studies on the problem were initiated only at the beginning of 21^st^ century. The research, carried out by us previously and already published, demonstrated that a significant amount of morphologic, physiologic and genetic differentiation exists between the isolated population of *B. laevigata* from the calamine areas near Olkusz (S Poland) and the nearest mountainous populations located in the Tatra Mountains and belonging to subsp. gracilis. We determined that the calamine population differs from subsp. gracilis in having light-green, thin rosette leaves densely covered by simple epidermal hair (Wierzbicka and Pielichowska 2004) (Fig. 2A–C). Our research has also demonstrated that the calamine morphotype has smaller seeds (as compared to subsp. gracilis) and shows an intense formation of daughter rosettes through vegetative reproduction (Fig. 2D). Furthermore, we identified the presence of pronounced physiological differences, including increased tolerance to Zn, Pb and Cd present in the calamine population (Wierzbicka and Pielichowska 2004). Our previous research showed that this differentiation is not merely a result of phenotypic plasticity. The phenotypic differentiation was stable and preserved when plants were grown in the greenhouse under standard conditions (Wierzbicka and Pielichowska 2004) (Fig. 2). Genetic analyses, carried out using Amplified Fragment Length Polymorphisms (AFLPs), showed that a strong genetic differentiation exists between calamine populations and the nearest natural populations located in the Western Carpathians (Wasowicz et al. 2014) and there are no signs of gene flow between these two areas. No evident signs of a bottleneck effect were evidenced by AFLP (Wasowicz et al. 2014). The DW Index (Schönswetter and Tribsch 2005), measuring the genetic divergence, was high in the calamine population, suggesting their long-term isolation (Wasowicz et al. 2014).

The paleobotanical study carried out in the near vicinity clearly suggests that calamine populations in the area of Olkusz could have originated before the Last Glacial Maximum (LGM) from local, interglacial populations. The presence of the species in the region has been determined by Szafer (1930), who, during his research in Ludwinow (about 40 km SE from Olkusz), found fossil siliques of *Biscutella laevigata* dating back to the Weichselian glaciation in Northern Europe (115–11.7 kyr BP, mainly corresponding to the Würm glaciation in the Alps and the Valdai glaciation in Eastern Europe).

A recently published study, carried out on local populations in southern Poland and focused on population genetic structure using nine nuclear micro-satellite loci (Babst-Kostecka et al. 2014), fully agrees with the paleobotanical data and our AFLP results (Wasowicz et al. 2014). Babst-Kostecka et al. (2014) concluded that the local calamine population in the vicinity of Olkusz originated as a result of an old vicariance predating the Last Glacial Maximum.

All these findings have led us to propose a hypothesis that the calamine population of *B. laevigata* from Olkusz Ore Bearing Region is a descendant of an ancient relict population that, through development of a series of adaptations to heavy metal stress, colonised natural calamine areas in the vicinity of Olkusz and, subsequently (when natural calamine sites were destroyed due to mining activities), also secondary sites (Wasowicz et al. 2014). Taking the above-mentioned differences into account, we argue that the morphotype from calamine areas in the vicinity of Olkusz should be formally recognised at subspecies level.

## Supplementary Material

XML Treatment for Biscutella laevigata L. subsp. woycickii
